# Acid-Catalyzed Water Extraction of Two Polysaccharides from *Artemisia argyi* and Their Physicochemical Properties and Antioxidant Activities

**DOI:** 10.3390/gels8010005

**Published:** 2021-12-22

**Authors:** Yuan Ruan, Chaofei Niu, Pengzhan Zhang, Yanyan Qian, Xinxin Li, Li Wang, Bingji Ma

**Affiliations:** Department of Traditional Chinese Medicine, Henan Agricultural University, Zhengzhou 450001, China; ruanyuanmbj@163.com (Y.R.); niucfhsxx@163.com (C.N.); zhangpz0625@163.com (P.Z.); qian18838922613@163.com (Y.Q.); lixinxinqq1024@163.com (X.L.)

**Keywords:** *Artemisia argyi*, polysaccharides, physicochemical properties, antioxidant activities

## Abstract

In this study, two purified polysaccharide fractions, Artp1 and Artp2, were obtained using acid-catalyzed water extraction, and then purified by DEAE-52 cellulose and Sephadex G-200 column chromatography from the crude polysaccharides of *Artemisia argyi*. Their physicochemical properties were investigated by gel permeation chromatography (GPC), high-performance anion exchange chromatography (HPAEC), Fourier transform infrared (FT-IR), scanning electron microscope (SEM), thermal analysis, and methylation analysis. The average molecular weight (Mw) of Artp1 and Artp2 were estimated to be 42.17 kDa and 175.22 kDa, respectively. Monosaccharide composition analysis revealed that the Rha, Gal, and GalA occupied main proportion in Artp1 with the molar ratio of 25.1:24.7:40.4, while the Rha, Gal, Xly, and GalA occupied the main proportion in Artp2 with the molar ratio of 16.7:13.5:12.8:38.7. Due to the high yield and the relatively high carbohydrate content, the Artp1 was determined by the methylation analysis and NMR. The results of Artp1 indicated that 1,4-Gal*p*A and 1,2,4-Rha*p* formed the backbone with some 1,2-Rha*p*, 1,3-Gal*p*, and 1,6-Gal*p* in the backbone or the side chains. Artp1 and Artp2 exhibited effective antioxidant activities by DPPH radical scavenging assay and hydroxyl radical scavenging assay in a dose-dependent manner. These investigations of the polysaccharides from *A. argyi*. provide a scientific basis for the uses of Artp1 and Artp2 as ingredients in functional foods and medicines.

## 1. Introduction

*Artemisia argyi* (*A. argyi*), known as “Aicao”, is a perennial herbaceous plant widely distributed in China for a long history. It is a popular and traditional medicinal and edible plant to treat dysmenorrhea, abdominal pain, inflammation, microbial infections, hepatitis, malaria, cancer, and circulatory disorders [1,2,3]. There are four species of famous *A. argyi* recorded in China, namely Northern *A. argyi*, Hai *A. argyi*, Qichun *A. argyi*, and Qi *A. argyi*, which are known as genuine herbs having high quality. Among them, North *A. argyi*, distributed in Tangyin County, Henan Province, is also called “Jiu Tou Xian Ai” because of the nine veins in its leaf. Due to its excellent quality and profoundly cultural heritage of the origin, North *A. argyi* is a “national geographical indication protection production of China” [4]. Only in Tangyin County, the planting area is about 1000 hectare in 2018 (http://www.rmzxb.com.cn/c/2018-05-08/2047335.shtml, accessed on 8 May 2018). It has been found that the chemical compositions in *A. argyi* can be classified into volatile essential oil and nonvolatile compounds. The latter, mainly comprised of flavonoids, organic acids, coumarins, terpenoids, and polysaccharides, afford various health-promoting potentials [5].

In past decades, polysaccharides isolated from natural sources, especially Chinese medicine plants, have been widely studied, owing to its various biological functions and low toxicity [3]. Previous studies showed that polysaccharide, which is one of the main components of *A. argyi*, had many good biological activities, including antitumor, immunomodulatory, and antioxidant activities. However, these cultivated species come from the Hubei province and the Anhui province [3,6]. The components of *A. argyi* cultivated in different regions were different. Up till now, there are few studies on polysaccharides from northern *A. argyi*, especially in purification and structure. The antioxidant activity of polysaccharide is closely related to their chemical properties and structures, such as monosaccharide composition, molecular weight, and sugar chain structure. It is necessary to determine the structure of polysaccharide for developing polysaccharide as natural antioxidants.

The traditional hot water extraction requires long extraction times and high temperatures [7]. According to the pre-experimental result, the yield and content of polysaccharide were not very well obtained by hot extraction. The acid can help eliminate the physical and chemical effects between the cell wall of *A. argyi*, and more polysaccharides can dissolve from cells into solution. In the current study, two fractions (named Artp1 and Artp2) were obtained by the acid-catalyzed water extraction and purified. Then, the chemical composition, preliminary and advanced structural characteristics, and antioxidant activities of Artp1 and Artp2 were comprehensively explored. The aim of this study was to characterize and compare the polysaccharides in their physicochemical and antioxidant activities, and offer a scientific foundation for their applications in functional food and medical fields.

## 2. Results and Discussion

### 2.1. Yields and Chemical Compositions

**Figure 1:**
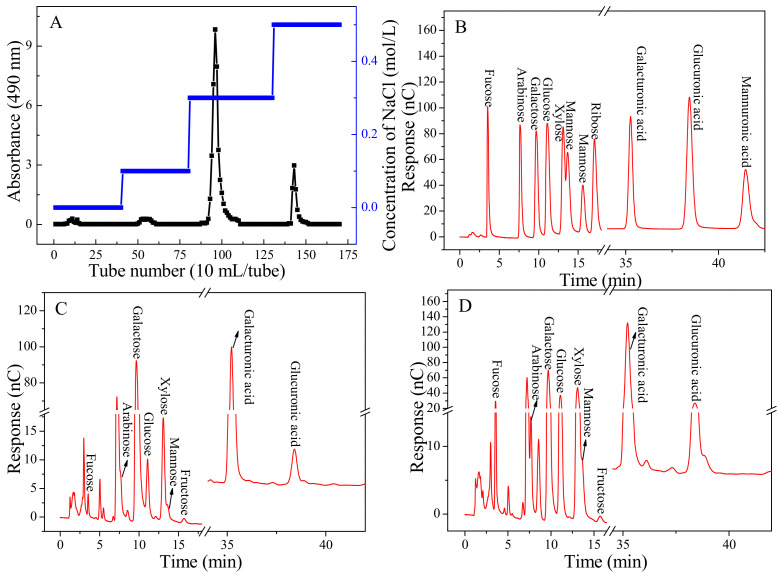
The DEAE-52 cellulose column chromatograms of Artp1 and Artp2 (**A**), HPAEC-PAD chromatogram profiles of standard monosaccharide mixture solution (**B**), and the monosaccharides from Artp1 (**C**) and Artp2 (**D**).

The pre-treatment result indicated that the yield of *A. argyi* polysaccharide was not very well obtained by traditional hot water extraction. This reason could be attributed to the saccharide structural properties, which had high cellulose and hemicellulose contents [8]. The dried *A. argyi* leaves were fully extracted using dilute acid and the crude polysaccharide was obtained. Its yield was 14.06% of raw material powder, which is higher than that extracted by hot water [3]. This could be attributed to the reason that the structure of the cell wall could be broken down, causing polysaccharides in the cell to be diffused into the solvent. The crude polysaccharide was purified by DEAE-52 cellulose column chromatography, and Artp1 and Artp2 were obtained by eluting with 0.3 mol/L and 0.5 mol/L NaCl, respectively (Figure 1). The Artp1 and Artp2 were further collected, concentrated, and purified by the sephadex G-100 gel filtration chromatography, and their yields were 6.63% and 1.42% of raw material powder, respectively.

The chemical components of Artp1 and Artp2 are summarized in Table 1. The carbohydrate contents of Artp1 and Artp2 were 574.30 mg/g and 448.17 mg/g, respectively. The uronic acid contents of both polysaccharides were 373.93 mg/g and 391.04 mg/g, respectively. These results of 37.39% and 39.10% (shown as percentages) were higher than those reported from *A. argyi* leaves (6.9%), which suggested that they might have good bioactivity [3]. Protein content of Artp1 and Artp2 were 10.67 mg/g and 25.54 mg/g, respectively. The phenolic contents of Artp1 and Artp2 were 0.2 and 1.35 mg/g, respectively. This result suggested that the extraction and purification of polysaccharides from *A. argyi* were successful.

### 2.2. Molecular Weight and Monosaccharide Composition

The weight-average molecular weight (Mw) distribution of Artp1 and Artp2 were analyzed by HPGPC and the results are shown in Table 2 and Figure S1. The Mw values of Artp1 and Artp2 were estimated to be 42.17 kDa and 175.22 kDa, respectively (Table 2). The polydispersity index (PDI) of Artp1 and Artp2 were 1.49 and 1.51, respectively. It indicated that relatively narrow distributions of polysaccharide chains in the sodium phosphate buffer solution. The Mw values of Artp1 and Artp2 were significantly higher than that of 5169-9171 Da obtained by Lan et al. and Bao et al. [3,6]. It might be due to the different extraction processes and the different sources of samples.

The structural properties and biological activities of plant polysaccharides are closely related to their monosaccharide composition [9]. The monosaccharide compositions of Artp1 and Artp2 were analyzed by HPAEC, and the results are presented in Figure 1 and Table 3. As shown in Figure 1B, the peaks of 11 standard monosaccharides were well-proportioned and the interval between peaks was obvious, which indicated that each standard monosaccharide could be separated well in liquid chromatography. The data showed that the monosaccharide types of the two fractions were the same, mainly composed of 8 monosaccharides: fucose (Fuc), arabinose (Ara), rhamnose (Rha) galactose (Gal), glucose (Glc), xylose (Xyl), mannose (Man), galacturonic acid (GalA), glucuronic acid (GlcA), and mannuronic acid (ManA). The molar ratio of 10 monosaccharides in Artp1 were 0.5:1.3:25.1:24.7:0.2:5.2:0.8:40.4:0.2:1.6, respectively, and that in Artp2 were 3.8:1.6:16.7:13.5:5.3:12.8:1.6:38.7:4.4:1.6, respectively. The result indicated that Artp1 and Artp2 were mainly composed of Rha, Gal, GalA, and Rha, Gal, Xly, and GalA, respectively. Both of Artp1 and Artp2 belong to acid heteropolysaccharides. The result of monosaccharide composition was different from that of the other polysaccharides obtained from *A. argyi* in previous studies. N-d-glucosamine, glucose, and mannose were the main composition of *A. argyi* (Qi Ai, Anguo City) polysaccharide. A small amount of Gal, Rha, Ara, Xyl, and Rib also existed in *A. argyi* (Qi Ai, Anguo City) polysaccharide. Man and Glc were the main compositions of polysaccharides obtained from *A. argyi* cultivated in Anhui, and little Xly, and Ara were also detected in them. Fuc, GalA, and GlcA in Artp1 and Artp2 have been newly found [3,6]. It indicated that the structures of Artp1 and Artp2 were different from polysaccharides of *A. argyi*, as reported previously. This indicated that the potentially different bioactivities of *A. argyi* polysaccharides could be obtained.

### 2.3. FT-IR

The structure features and components of polysaccharides can be inferred based on the characteristic absorption caused by vibration induced molecular dipole moment or charge distribution [10]. The infrared spectra between 400 cm^−1^ and 4000 cm^−1^ of Artp1 and Artp2 were shown in Figure S2. Obviously, the FT-IR spectra of Artp1 and Artp2 are similar, and the reason could be attributed to the similar monosaccharide compositions of two fractions. The strong and broad characteristic peaks at around 3416 cm^−1^ and 3420 cm^−1^ were attributed to the hydroxyl group. The weak bands at around 2925 cm^−1^ and 2924 cm^−1^ in two fractions can be attributed to C-H groups. The absorption peaks at around 1613 cm^−1^ and 1420 cm^−1^ indicated the presence of carboxyl groups, and that Artp1 and Artp2 contained uronic acid [3]. This was consistent with the results of monosaccharide compositions. The region between 1200–950 cm^−1^ of each polysaccharide was the so-called fingerprint region. The position and intensity of the bands within the region were specific for each polysaccharide, and this could provide the structural information [11]. The intense peaks at 1042 cm^−1^ and 1092 cm^−1^ were attributed to the glycosidic linkage C-O-H and C-O-C stretching vibration. Meanwhile, the peak at 1042 cm^−1^ was characteristic of rhamnose [2]. The characteristic absorptions at around 822 cm^−1^ and 895 cm^−1^ were identified in Artp1 and Artp2, indicating the existence of α-configuration and β-configuration of the sugar units [12].

### 2.4. Thermal Analysis

Thermal properties of polysaccharides are usually used to evaluate the weight loss, chemical changes, and thermal behavior of complex polysaccharides.

**Figure 2:**
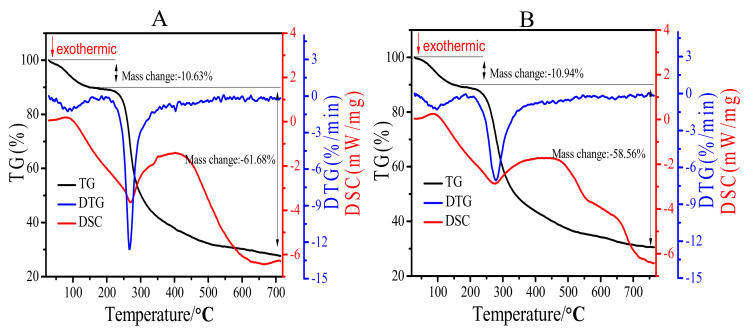
TG, DTG and DSC curves of Artp1 (**A**) and Artp2 (**B**).

As shown in Figure 2, there were two different stages shown in DTG profiles of Artp1 (Figure 2A) and Artp2 (Figure 2B). The first mass loss of Artp1 (10.63%) and Artp2 (10.94%) occurred within the temperature range of 30 °C to 180 °C and the major peaks were at 80.6 °C and 84.6 °C, respectively. The reason was attributed to the loss of free and bound water in Artp1 and Artp2. At the second stage between 180 °C and 700 °C, rapid and maximum weight loss were observed for Artp1 (61.68%) and Artp2 (58.56%), which was attributed to a depolymerization and decomposition reactions [13]. These results suggested that Artp1 and Artp2 were relatively stable below 270 °C.

The DSC curves of Artp1 and Artp2 show exothermal or endothermal changes with increased temperature. The first endothermic peaks of Artp1 and Artp2 were mainly the loss of the bound water or partial decomposition. The main exothermic peaks for Artp1 and Artp2 appeared at 270.0 °C and 272.6 °C, respectively, which indicated that thermal degradation of the polysaccharide. This was in accordance with the DTG result. The thermal properties elucidate that Artp1 and Artp2 were highly thermostable below 270 °C.

### 2.5. Methylation Analysis

After methylation treatment, all the free OH groups are completely methylated. The individual peaks of the partially methylated alditol acetate (PMAA) and fragmentation patterns were identified by their retention times in GC-MS and mass spectra patterns in the literatures. Based on this analysis of PMAA, the molar percent ratios of various types of sugar residues and the linkage patterns of Artp1 are shown in Table 4. The result indicated that the rhamnose residues were present as 1,4-linked and 1,2,4-linked Rhap residues, and the galacose residues were present as terminal, 1,3-linked, and 1,6-linked Galp residues. However, the galacturonic acid residues were present as 1,4-linked and 1,3,4-linked GalpA residues. It indicated that Artp1 is highly branched and that the side chains were mostly terminated by Galp residues. The data from the methylation analysis suggested that Artp1 is mainly composed of a 1,4-GalpA and 1,2,4-Rhap backbone with some 1,3-Galp and 1,6-Galp bonds which could be in the backbone or the side chains. The results agreed with monosaccharide composition analysis, which demonstrated that the main chain of Artp1 contained mainly rhamnose, galactose, and galacturonic acid.

### 2.6. Nuclear Magnetic Resonance (NMR) Analysis

**Figure 3:**
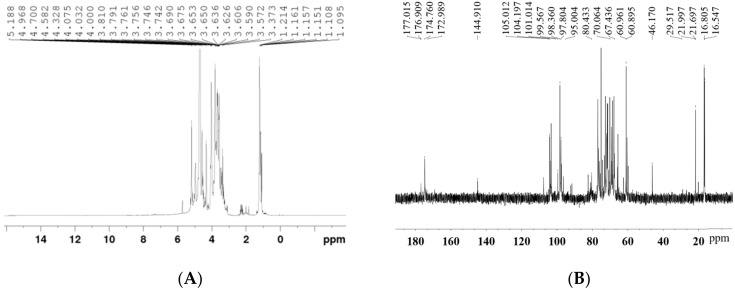
The ^1^H (**A**) and ^13^C (**B**) NMR spectra of Artp1.

The ^1^H and ^13^C NMR spectra of Artp1 are shown in Figure 3. The ^1^H NMR spectrum of the polysaccharide was mostly crowded in a narrow region ranging from 3 to 5 ppm. The H2-H6 signal concentration region between 3.1 and 4.5 ppm is difficult to assign [14]. The anomeric hydrogen of the polysaccharide appears between 4.3 and 6.0 ppm, with 4.3–4.8 ppm being the β-confguration and 4.9–5.2 ppm being the α-confguration [15]. It indicated that the existence of both α- and β- configurations in Artp1.

In the ^13^C NMR spectrum (Figure 3B), the signals from the anomeric carbons appeared between 97.80 and 104.19 ppm, which indicated that there were both α (δ 95–101 ppm) and β (δ 101–105 ppm) anomeric configurations existing in Artp1 [16]. The signals at 1.79–2.23 ppm were assigned to acetyl groups in the ^1^H spectrum, and the signal between 22.0–29.5 ppm represented acetyl groups of the monosaccharide units in the ^13^C spectrum. The absence of these signals indicated the absence of the acetyl groups of Artp1. Two peaks at 1.15 ppm and 1.21 ppm for Artp1 were from proton resonances of methyl groups in Rha. The corresponding resonances in the ^13^C spectrum were at 16.80 ppm and 16.54 ppm. The anomeric proton signals occurring at 4.69 ppm and 4.96 ppm were designated as 1,3-β-d-Galp and T-Galp, respectively, and the corresponding anomeric carbon signals in ^13^C NMR spectrum were 104.19 ppm and 97.80 ppm. Two anomeric proton signals appeared at 5.19 and 5.22 ppm, and could be assigned to H-1 of 1,2,4-α-L-Rhap and 1,2-α-L-Rhap, respectively. The anomeric carbon signals at 99.56/98.36 ppm were attributed to the C-1 of 1,2,4-α-L-Rhap and 1,2-α-L-Rhap [17]. In the ^13^C spectrum, the signals in the region for the resonances of carbonyl groups (carboxyl and ester carbonyls) at 173–177 ppm corresponded to the C-6 of unesterified or esterified galacturonic acid units. In addition, the signals at 80.43 ppm and 67.4–70.0 ppm confirmed the existences of (1 → 3/4) and (1 → 6) glycosidic linkages, respectively [18]. The results of NMR were consistent with the results of monosaccharide composition analysis and methylation analysis.

### 2.7. SEM Analysis

**Figure 4:**
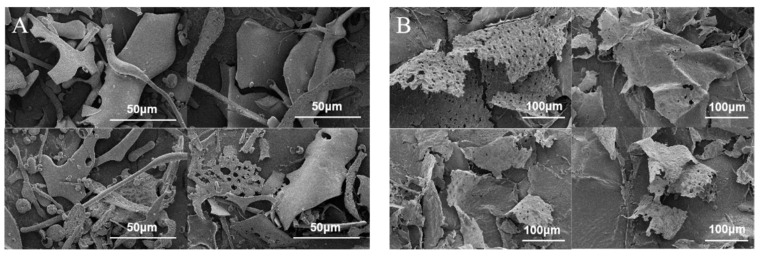
Scanning electron micrograph images of Artp1 and Artp2. (**A**) Artp1, ×3000 and ×2000, 50 μm; (**B**) Artp2, ×1000, 100 μm.

Scanning electron micrographs (SEM) is a qualitative tool for analyzing the surface morphology of plant polysaccharides [9]. Scanning electron micrograph of Artp1 and Artp2 is shown in Figure 4. Furthermore, the structural dimensions of the two samples were analyzed using Nano Measurer 1.2 software. The results showed a significant difference in the shape and size of the two polysaccharides. As shown in Figure 4A, the morphology of Artp1 was significantly different from that of the Artp2, and the surface of Artp1 was smooth and showed a large sheet structure with a width of 12.05–55.40 μm [19]. In addition, Artp1 contained the unevenly spherical shapes with a pore size of 0.78–12.03 μm, unevenly netted structures with the diameter of 0.94–5.75 μm and a rod-shaped structure with the length of 18.79–267.34 μm. However, the Artp2 (Figure 4B) had thin slice-shape structures with curled surface and wrinkles, and a rough surface. This could be attributed to molecular aggregation [20]. The lengths of slice shape structures were among 155.66–410.23 μm and the widths of them were 35.26–281.81 μm. The difference in the morphologies of Artp1 and Artp2 may be attributed to the differences in their molecular structures, molecular weight, and monosaccharide composition [21].

### 2.8. Antioxidant Activities

#### 2.8.1. DPPH Radical Scavenging Assay

**Figure 5:**
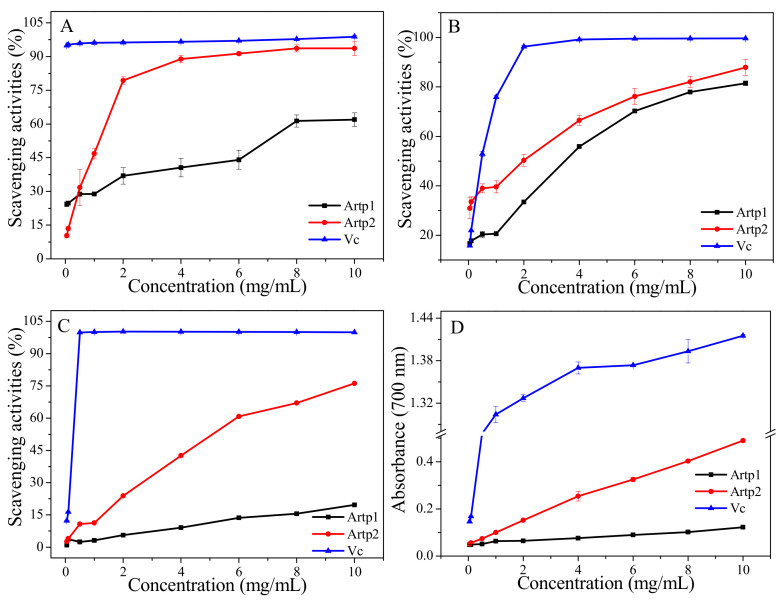
Antioxidant activities of Artp1 and Artp2: (**A**) DPPH radical scavenging activity; (**B**) hydroxyl radical scavenging activity; (**C**) ABTS radical scavenging activity; (**D**) reducing power.

The DPPH radical scavenging is based on the hydrogen-donating ability of antioxidants. Due to the advantages of simple operation, fast speed, and high sensitivity, it is widely used to test the antioxidant capacities of various antioxidant samples [19,22]. The DPPH radicals’ scavenging rate of vitamin C (Vc) was stable at about 94–99% in the test concentration range. DPPH radicals’ scavenging activities of Artp1 and Artp2 are showed in Figure 5A. The two fractions showed concentration-dependent scavenging activities against the DPPH radical. Artp2 appeared to be more effective than that of Arp1 with the maximum inhibition rate of 88.29–93.65% in the range of 4–10 mg/mL. The maximum DPPH radicals’ scavenging activity of Artp2 was close to Vc control. In addition, Artp2 exhibited good DPPH radicals’ scavenging activity with a maximum inhibition rate of 61.96% at 10 mg/mL. The difference in DPPH radicals’ scavenging activities of Artp1 and Artp2 could be related to the difference in the monosaccharide compositions, molecular weight, glycosidic linkage, and solubility of the polysaccharides [9].

#### 2.8.2. Hydroxyl Radical Scavenging Assay

Hydroxyl radicals are harmful-free radicals generated in the human body. They not only react with carbohydrates, proteins, lipids, and nucleic acids inside the body and hereby cause cell damage, but also promote the generation of reactive oxygen species, which could induce severe oxidative damage of different tissue [9,23]. The ability to deactivate hydroxyl free radicals is an important indicator used to evaluate the antioxidant activity of a substance or molecule. Hydroxyl radicals scavenging activities of Artp1 and Artp2 were determined and compared to that of the positive control (Vc). It can be seen from Figure 5B that the hydroxyl radical scavenging activities of Artp1 and Artp2 increased with the increase in their concentration. However, there was no significant difference on scavenging activities between Artp1 and Artp2 at the test concentration range. At a concentration of 10 mg/mL, the maximum hydroxyl radical scavenging activities of Artp1 and Artp2 were 81.47% and 87.87%, respectively, which were close to that of Vc. The activity of Artp2 was slightly stronger than that of Artp1, and this could be attributed to the high contents of protein and uronic acid in Artp2. This result was consistent with the findings of previous studies [24,25].

#### 2.8.3. ABTS Radical Scavenging Assay

The ABTS radical scavenging activity assay was shown to be simple and quick in operation. It has been frequently used for measuring the antioxidant capacities of foods and natural products [26]. Figure 5C shows the ABTS radical scavenging activities of Artp1 and Artp2. The ABTS radical scavenging activities of Artp1 and Artp2 were concentration-dependent in the concentration range. ABTS radicals scavenging activities of the positive control (Vc) reached 100% at a concentration of 0.5 mg/mL. The ABTS radical scavenging activity of Artp2 (10 mg/mL) reached 76.19%, indicating the efficient antioxidant activity ofArtp2. This result is similar with the previous study, which indicated that a polysaccharide extracted from *A. argyi* displayed good ABTS radicals scavenging effects [27]. However, Artp2 showed significantly higher ABTS scavenging activity (76.19%) than that of Artp1 (19.64%) at 10 mg/mL, which might also be due to the fact that Artp2 contains a higher amount of phenolic, protein, and uronic acid. These results indicated that Artp2 had strong scavenging power for ABTS radical and could be explored as novel potential antioxidant.

#### 2.8.4. Reducing Power

Fe^3+^ reduction can be used as an indicator of electron-donating activity, reflecting an important mechanism of polysaccharide antioxidant action [28]. In this assay, reducing the power of antioxidants was reduced by the decrease in the Fe^3+^–ferricyanide complex to the ferrous form, and was determined at 700 nm. As observed in Figure 5D, the reducing power of Artp1, Artp2, and Vc increased with their concentrations, which is in agreement with previous reports of antioxidant potential of polysaccharides extracted from *Zizyphus Jujuba cv. Jinsixiaozao* [24]. At the concentration of 10 mg/mL, the reducing capacities of Artp1, Artp2, and Vc were 0.12, 0.49, and 1.41, respectively. The results indicated that the reducing power of Artp2 was significantly higher than that of Artp1 at the same concentration. The results showed that Artp2 had potential antioxidant properties. Reducing properties are generally associated with the presence of reductones, which could react with certain precursors of peroxide, thus preventing peroxide formation [24].

## 3. Conclusions

Two purified non-starch polysaccharide fractions, Artp1 and Artp2, were successfully obtained using DEAE-52 cellulose and Sephadex G-200 column chromatography from the crude polysaccharides of *A. argyi* with acid water. These fractions’ structures were investigated by GPC, HPAEC, FT-IR, SEM, thermal analysis, and methylation analysis. The average molecular weight (Mw) of Artp1 and Artp2 were estimated to be 42.17 kDa and 175.22 kDa, respectively. Monosaccharide composition analysis revealed that the Rha, Gal, and GalA occupied the main proportion in Artp1 with the molar ratio of 25.1:24.7:40.4, while the Rha, Gal, Xly, and GalA occupied the main proportion in Artp2 with the molar ratio of 16.7:13.5:12.8:38.7. Due to the high yield and the relatively high carbohydrate content, the Artp1 was determined by the methylation analysis and NMR. The results of Artp1 indicated that 1,4-GalpA and 1,2,4-Rhap formed the backbone with some 1,2-Rhap, 1,3-Galp, and 1,6-Galp in the backbone or the side chains. Artp1 and Artp2 exhibited effective antioxidant activities by the DPPH radical scavenging assay and hydroxyl radical scavenging assay in a dose-dependent manner. These investigations of the polysaccharides from *A. argyi* provide a scientific basis for the use of *A. argyi*, particularly as an ingredient in functional foods and medicines. Artp1 and Artp2 have the potential to be explored as novel antioxidant products. The more valuable functions of Artp1 and Artp2 need to be explored further.

## 4. Materials and Methods

### 4.1. Materials and Reagents

*A. argyi* was collected at Tangyin County, China in Aug 2018. Dialysis bag (500–1000 Da and 3500 Da) was purchased from Shanghai Yuanye Bio-Technology Co., Ltd. (Shanghai, China). DEAE-52 cellulose column and AB-8 resin were purchased from Beijing Solarbio Science & Technology Co., Ltd. (Beijing, China). Sephadex G-200 column was purchased from Sigma Chemical Co. (St. Louis, MO, USA). Ascorbic acid (Vc) was purchased from Paini Chemical Reagent Factory (Zhengzhou, China). Dextrans of different molecular weights were purchased from Beijing Solarbio Science & Technology Co., Ltd. (Beijing, China). The standard monosaccharides were purchased from Sigma Chemical Co. (St. Louis, MO, USA). DPPH (1,1-Dipenyl-2-picrylhydrazyl Free Radical) was purchased from Tokyo Chemical Industry Co., Ltd. (Tokyo, Japan). The reagents and solvents in the study were of analytical grade (AR).

### 4.2. Extraction the Crude Polysaccharides from A. argyi

The leaves of *A. argyi* were thoroughly washed with water, dried at 45 °C, and then powdered with the pulverizer. The powder was extracted with 95% ethanol at room temperature for 24 h twice. After filtering, the residues were air-dried, and then the residue was further extracted with 0.25 M HCl in a water bath (80 °C) with continuous stirring for 3 h two times. After being combined and neutralized with 1 M NaOH, the above extracts were concentrated. Afterwards, the above extract was precipitated by adding ethanol until the concentration of ethanol reached 80% at 4 °C overnight, followed by centrifugation at 4000 rpm for 10 min. After being washed successively with ethyl acetate and acetone, the precipitate was dissolved in deionized water and subsequently dialyzed against deionized water for 48 h with a 3500-Da dialysis bag. Next, the dialysates were concentrated and then their protein contents were degraded using Sevage reagent, followed by de-colorization using D-101 resin. Then, the above solution was lyophilized, which yielded the crude *A. argyi* polysaccharides.

### 4.3. Purification of Polysaccharide

The crude polysaccharides were dissolved in distilled water and applied to a DEAE-52 cellulose column (2.6 × 30 cm), followed by step-wise elution with distilled water and NaCl solutions of 0.1, 0.3, and 0.5 M at a flow rate of 1.0 mL/min. After concentrated, dialyzed with a 3500-Da dialysis bag, and lyophilized, the fractions eluted with distilled water were further purified on a Sephadex G-200 column (2.6 × 100 cm) with the elution of distilled water at a flow rate of 0.6 mL/min, leading to a finally purified polysaccharide that was designated as Artp1 and Artp2 (eluted by 0.3 M and 0.5 M NaCl solutions, respectively.).

### 4.4. Characterization of Artp1 and Artp2

#### 4.4.1. Chemical Composition Analysis

Having been established in our laboratory, methods were employed to determine and calculate the contents of carbohydrate, uronic acid, protein, and phenols [23]. The standards of curves were obtained by various concentrations of glucose, galacturonic acid, bovine serum albumin, and gallic acid.

#### 4.4.2. Molecular Weight Detection and Monosaccharide Composition Analysis

Gel permeation chromatography (GPC) was used to determine the molecular weights of Artp1 and Artp2 on a Shimadzu SPD-20A HPLC system equipped with a SUGAR KS-805 column (300 × 8.0 mm, Shodex, Japan) [7]. Briefly, 1.5 mg/mL of purified sample was dissolved in 0.02 M sodium phosphate buffer (pH 6.8) and percolated through 0.45 μm of millipore filter, and then applied to column (injection volume of 30 μL) with a flow rate of 1 mL/min at a column temperature of 55 °C. Preliminary calibration of the column was conducted using the Dextrans with different molecular weights (4320, 12,600, 73,800, 126,000, 289,000, and 1,000,000) The regression curve equation was as follows: LogMw = −1.1337Rt + 16.165 with a correlation coefficient of 0.9912.

The monosaccharide compositions of samples were determined by high-performance anion-exchange chromatography pulsed amperometric detection (HPAEC-PAD) (Dionex ICS-5000, Thermo Fisher, Waltham, MA, USA). Next, 5 mg of polysaccharide was dissolved in TFA and hydrolyzed at 121 °C for 2 h. Then, the hydrolyzed product was washed by evaporating at reduced pressure with methanol for 2–3 times. After this, the samples of hydrolyte were diluted and filtrated through 0.22 μm of hydrophilic membranes. The reaction products were analyzed with a Dionex™ CarboPac™ PA20 analytical column to elute with a mobile phase at 0.5 mL/min.

#### 4.4.3. Fourier Transform Infrared Spectroscopy Analysis (FT-IR)

The IR spectra of Artp1 and Artp2 were obtained using a Bruker Vector 22 spectrometer (Bruker, Rheinstetten, Germany). All samples were mixed with KBr powder, pressed into pellets at room temperature, and then recorded in the range of 4000 to 400 cm^−1^.

#### 4.4.4. Thermal Analysis

Thermal analysis of Artp1 and Artp2 was carried out according to the previously reported method [23]. Thermal analysis was performed on a thermogravimetric analysis instrument (Netzach, STA 449 F3 Jupiter, Germany), containing thermogravimetric analysis (TG), differential thermal gravity (DTG), and differential scanning calorimetry (DSC). Then, 3–5-mg samples were recorded at the range of 30–700 °C under nitrogen atmosphere with a heating rate of 10 °C/min.

#### 4.4.5. Methylation Analysis

The methylation analysis was determined according to the previous report and were analyzed by the Agilent 6890A-5975C system (Agilent Technologies Inc., Santa Clara, CA, USA), equipped with a BPX70 column [7]. Briefly, the mixture containing 1 mL of polysaccharide (3 mg/mL), 200 μL of MES (0.2 mol/L), and 200 μL of carbodiimide (500 mg/mL) was incubated at room temperature for 2 h. The sample was divided into two parts on average after 1 mL of 4 M imidazole was added. NaBH_4_ and NaBD_4_ were added into the two samples, respectively. The reactions were kept at room temperature for 3 h. Finally, the reactions were stopped with 200 μL of glacial acetic acid. Next, the reaction mixture was dialyzed for 48 h using against deionized water (Mw cut-off 3500 Da). Then, the sample was dried using by freeze-drying and dissolved again with 0.5 mL of DMSO. Then, 50 μL of DMSO/NaOH solution (120 mg/mL) was added and incubated for 30 min, and then methylated by adding 30 μL of CH_3_I to incubate for 1 h (Add 3 times at a rate of 10 μL/10 min). Methylated samples were hydrolyzed with 2 M TFA at 121 °C for 4 h and converted into their corresponding alditols by reduction with NaBD_4_ and acetylated. The entire methylation analysis experiment was carried out in an absolute dry environment, and all reagents are free of moisture. After reduction and acetylation of the hydrolysates, the PMAA derivatives were analysed with a GC-MS system (6890A-5975C, USA).

#### 4.4.6. Nuclear Magnetic Resonance (NMR) and Scanning Electron Microscope (SEM) Analyses

The sample (20 mg) was dissolved in 0.55 mL of D_2_O and transferred into a 5-mm NMR tube. NMR spectra (^1^H NMR and ^13^C NMR) were analyzed by Bruker AVANCE III HD 500 NMR Spectrometer (Bruker Co., Billerica, MA, USA). The SEM (FEI Quanta 250 FEG) was used to observe the surface morphological characteristics of Artp1 and Artp2. The samples were sputtered with pt using a Cressington 108auto Sputter Coater, and the images were observed at a voltage of 3.0 kV.

### 4.5. Antioxidant Activities

#### 4.5.1. DPPH Radical Scavenging Assay

The DPPH radical scavenging activities in vitro of the polysaccharides were performed according to the previous report with some modifications [23]. Briefly, 1 mL of each sample solution with specific concentration (0.05, 0.1, 0.5, 1, 2, 4, 6, 8, and 10 mg/mL) was mixed with 1 mL of 0.05 mmol/L DPPH solution, respectively. For positive control, vitamin C (Vc) was used as antioxidant reactant. After dark reaction for 30 min, the absorbance of each mixture was measured at 517 nm with a microplate reader. The DPPH radical scavenging rate was calculated by the following formula:

$$\mathrm{DPPH}\;\mathrm{radical}\;\mathrm{scavenging}\;\mathrm{rate}\;(\%)=\left[1\;-\;\frac{A_{X}-A_{X0}}{A_{0}}\right]\times 100$$

*A*_0_ is the absorbance of the mixture without sample,

*A_X_* is the absorbance of sample solution group,

*A*_*X*0_ is the absorbance of the mixture without the DPPH solution.

#### 4.5.2. Hydroxyl Radical Scavenging Assay

The hydroxyl radical inhibitory activities of the above polysaccharide products were measured according to the previously reported method with slight modifications [23]. The concentrations used for each sample and reaction carrier are the same as described above in Section 4.5.1. For measurement, 50 μL of each sample solution was mixed with 50 μL of 4.5 × 10^−3^ mol/L FeSO_4_ solution and 50 μL of 4.5 × 10^−3^ mol/L salicylic acid 70% ethanol solution, sequentially. After homogeneous mixing, 100 μL of 6 × 10^−3^ mol/L H_2_O_2_ was added, and kept for 37 °C for 30 min. Vc was used as the positive control. Absorbance of the reaction solutions at 510 nm was recorded to calculate the hydroxyl radicals scavenging rates for each polysaccharide sample, using the following formula:

$$\mathrm{Hydroxyl}\;\mathrm{radical}\;\mathrm{scavenging}\;\mathrm{rate}\;(\%)=\left[1\;-\;\frac{A_{X\;}{-\;A}_{X0}}{A_{0}}\right]\;\times \;100$$

*A*_0_ is the absorbance of the mixture without sample;

*A_X_* is the absorbance of the sample solution group;

*A*_*X*0_ is the absorbance of the mixture without the H_2_O_2_ solution.

#### 4.5.3. ABTS Radical Scavenging Assay

ABTS radical scavenging activity was determined according to the reported method with some modifications [15]. The working solution was prepared by mixing equal volumes of 7 mmol/L ABTS solution and 2.45 mmol/L potassium persulphate. This meant that they could then be incubated for 16 h at room temperature in the dark. At the moment of use, the ABTS+ solution was diluted with PBS (pH 6.8) to an absorbance of 0.70 ± 0.05 at 734 nm. Briefly, 200 μL of samples with various concentrations (concentration is the same as 2.5.1) was mixed with 1.2 mL of the ABTS solution and mixed vigorously, respectively. The reaction mixture was allowed to stand at room temperature for 10 min, and the absorbance at 734 nm was recorded immediately. Vc was used as the positive control. The ABTS radical scavenging activity of the sample was calculated as follows:

$$\mathrm{ABTS}\;\mathrm{radical}\;\mathrm{scavenging}\;\mathrm{rate}\;(\%)=\left[1\;-\;\frac{A_{X\;}{-\;A}_{X0}}{A_{0}}\right]\;\times \;100$$

*A*_0_ is the absorbance of the mixture without sample;

*A_X_* is the absorbance of the sample solution group;

*A*_*X*0_ is the absorbance of the mixture without the ABTS solution.

#### 4.5.4. Reducing Power

The reducing power fractions were determined according to the previous method [15]. The concentrations used for each sample and reaction carrier are the same as described above in Section 4.5.1. Accurately, the mixture of the 0.1-mL sample solution, 0.2 mL of phosphate buffer solution (pH 6.8), and 0.1 mL of 1% (*w*/*v*) potassium ferricyanide solution was incubated at 50 °C for 20 min. This was then followed by swift cooling, and 1 mL of 10% (*w*/*v*) trichloroacetic acid solution was added. The mixture was centrifuged for 10 min at 4000 rpm. Next, 0.1 mL of the supernatant solution was diluted with 1.4 mL of distilled water, before adding 50 μL of 0.1% (*w*/*v*) FeCl_3_ solution at room temperature for 10 min. The absorbance of the mixture was measured at 700 nm. For comparison, Vc was used as the positive control sample. The absorbance value is the strength of the reducing power.

### 4.6. Statistical Analysis

The data were expressed as mean ± SD of three replicates and analyzed for variance (ANOVA) followed by Duncan’s multiple-range test. SPSS Statistics 21 was used for statistical analysis and the statistical significance of all tests was defined as *p* < 0.05.

## Figures and Tables

**Table 1:** The chemical compositions of Artp1 and Artp2.

Samples	Artp1	Artp2
Carbohydrate contents (%)	57.43 ± 3.25	44.82 ± 2.52
Uronic acid content (%)	37.39 ± 4.46	39.10 ± 2.64
Protein contents (%)	1.07 ± 0.01	2.56 ± 0.34
Phenolic contents (%)	0.02 ± 0.005	0.14 ± 0.022

**Table 2:** The molecular weights of Artp1 and Artp2.

Molecular Weight (kDa)	Artp1	Artp2
Weight-average molecular weight (Mw)	42.17	175.22
Number-average molecular weight (Mn)	28.19	115.95
Polymer dispersity index (PDI)	1.49	1.51

**Table 3:** The monosaccharide compositions of Artp1 and Artp2.

Monosaccharide Composition (Molar Ratio %)	Artp1	Artp2
Fucose (Fuc)	0.5	3.8
Arabinose (Ara)	1.3	1.6
Rhamnose (Rha)	25.1	16.7
Galactose (Gal)	24.7	13.5
Glucose (Glc)	0.2	5.3
Xylose (Xyl)	5.2	12.8
Mannose (Man)	0.8	1.6
Galacturonic acid (GalA)	40.4	38.7
Glucuronic acid (GlcA)	0.2	4.4
Mannuronic acid (ManA)	1.6	1.6

**Table 4:** Methylation analysis and mode of linkage of Artp1.

Methylated Sugar	Molar Ratio (%)	Linkage Type
3,4-Me_2_-Rhap	8.32	2-Rhap
3-Me_1_-Rhap	17.68	2,4-Rhap
2,3,4,6-Me_4_-Galp	31.53	t-Galp
2,4,6-Me_3_-Galp	1.51	1,3-Galp
2,3,4-Me_3_-Galp	1.41	1,6-Galp
2,3,6-Me_3_-GalAp	28.27	1,4-GalAp
2,6-Me_2_-GalAp	1.61	1,3,4-GalAp

## Data Availability

Not applicable.

## Supplementary Materials

The following are available online at https://www.mdpi.com/article/10.3390/gels8010005/s1, Figure S1: The molecular weight distribution curves of Artp1 and Artp2, Table S2: FT-IR spectra of Artp1 and Artp2.
