# Supplementary material for: Acid-Catalyzed Water Extraction of Two Polysaccharides from Artemisia argyi and Their Physicochemical Properties and Antioxidant Activities
Source: Gels. 2021 Dec 22;8(1):5. doi: 10.3390/gels8010005 (PMC8774486; doi:10.3390/gels8010005)
Supplement: Supplementary file 1 [file gels-08-00005-s001.zip › gels-1481784-supplementary.pdf]

gels

Article

Acid-Catalyzed Water Extraction of Two Polysaccharides from *Artemisia argyi* and Their Physicochemical Properties and Antioxidant Activities

Yuan Ruan, Chaofei Niu, Pengzhan Zhang, Yanyan Qian, Xinxin Li, Li Wang * and Bingji Ma *

Department of Traditional Chinese Medicine, Henan Agricultural University, Zhengzhou 450001, China; ruanyuanmbj@163.com (Y.R.); niucfhsxx@163.com (C.N.); zhangpz0625@163.com (P.Z.); qian18838922613@163.com (Y.Q.); lixinxinqq1024@163.com (X.L.)

* Correspondence: wanglihuina@163.com (L.W.); mbj12345@henau.edu.cn (B.M.)

Supplementary Materials

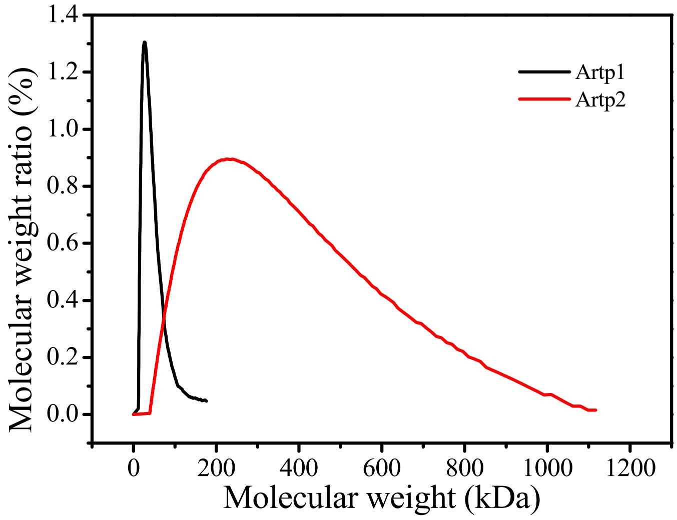

Figure S1. The molecular weight distribution curves of Artp1 and Artp2.

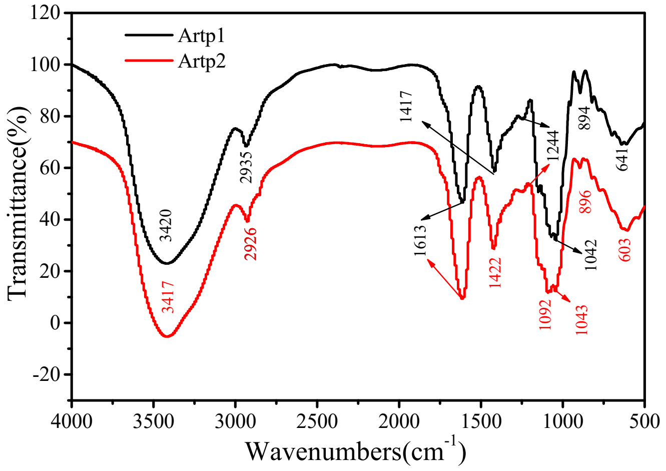

Figure S2. FT-IR spectra of Artp1 and Artp2.
